# Applications of Invertebrate Animal Models to Dimorphic Fungal Infections

**DOI:** 10.3390/jof4040118

**Published:** 2018-10-19

**Authors:** Junya L. Singulani, Liliana Scorzoni, Haroldo C. de Oliveira, Caroline M. Marcos, Patricia A. Assato, Ana Marisa Fusco-Almeida, Maria José S. Mendes-Giannini

**Affiliations:** School of Pharmaceutical Sciences, São Paulo State University (UNESP), Araraquara, São Paulo 14800-903, Brazil; junyadelacorte@yahoo.com.br (J.L.S.); liliscorzoni@yahoo.com.br (L.S.); haroldocdoliveira@gmail.com (H.C.d.O.); marcos_caroline@yahoo.com.br (C.M.M.); patricia.assato@gmail.com (P.A.A.); ana.marisa@uol.com.br (A.M.F.-A.)

**Keywords:** Dimorphic fungi, *Galleria mellonella*, *Caenorhabditis elegans*, *Acanthamoeba castellanii*, host-pathogen interactions, virulence, innate immunity, antifungal

## Abstract

Dimorphic fungi can be found in the yeast form during infection and as hyphae in the environment and are responsible for a large number of infections worldwide. Invertebrate animals have been shown to be convenient models in the study of fungal infections. These models have the advantages of being low cost, have no ethical issues, and an ease of experimentation, time-efficiency, and the possibility of using a large number of animals per experiment compared to mammalian models. Invertebrate animal models such as *Galleria mellonella*, *Caenorhabditis elegans*, and *Acanthamoeba* *castellanii* have been used to study dimorphic fungal infections in the context of virulence, innate immune response, and the efficacy and toxicity of antifungal agents. In this review, we first summarize the features of these models. In this aspect, the growth temperature, genome sequence, availability of different strains, and body characteristics should be considered in the model choice. Finally, we discuss the contribution and advances of these models, with respect to dimorphic fungi *Paracoccidioides* spp., *Histoplasma capsulatum*, *Blastomyces dermatitidis*, *Sporothrix* spp., and *Talaromyces marneffei (Penicillium marneffei)*.

## 1. Introduction

Use of animal experimentation for both scientific and medical areas of research is a fundamental step for validating *in vitro* results and understanding different processes in a complex organism [1]. Since 1959, the “3 Rs” principle (reduction, refinement, and replacement) suggested by Russell and Burch has been used to demonstrate concern regarding the ethical issues and animal welfare during animal experimentation. The 3Rs strategy motivates the Reduction of animals during experimentation, which could be achieved by well-designed experiments and statistical tools to obtain the highest amount of information from the experiment; the Refinement of procedures aiming to minimize animal suffering; and the Replacement of higher animals with other models or methodologies [1,2,3,4]. Some authors have included a 4th R, namely, Responsibility, which was added in 1995 and is related to honesty, and scientific rightness regarding the use of animals in research [4,5].

Dimorphic fungi are species that can switch morphology and these microorganisms can be mostly found in the yeast form during infection and as hyphae in the environment [6]. *In vivo* assays in mycology are useful to compare virulence between isolates, to analyze pathogenesis of infections, to study the efficacy of new compounds, and the immune response [6,7]. Considering fungal *in vivo* assays, including dimorphic fungi, the gold standard is represented by mammals as the murine model [6,7,8,9]. However, laws and acts of incentive control the use of these animals for experiments [1]. Although murine models have been recommended for *in vivo* experiments, they present some disadvantages such as high costs, time consuming protocols, and requirement of suitable space, equipment, and training for the procedures [2,4].

Based on this, the scientific society has developed different methodologies to apply the 3 Rs principles such as *in silico* analysis, cell and tissue culture, and invertebrate animal models [2]. Invertebrates such as *Galleria mellonella*, *Caenorhabditis elegans*, and *Acanthamoeba castellanii* have been successfully used for *in vivo* assays, including in the study of dimorphic fungi. These models are easier to develop, time-efficient, cost-efficient, and offer the possibility of using a large number of animals per experiment compared to mammalian models [10,11]. There are published protocols for maintaining these animals in laboratory conditions and the procedures to perform experiments [12,13]. Moreover, it is possible to observe a good correlation with mammalian models [14,15]. It is important to highlight that each model presents advantages and disadvantages, and sometimes, more than one model is necessary to obtain reliable results [16]. Thus, although invertebrate models are useful for infection studies and compound screening, complete substitution of mammalian models is still not recommended [4]. This review describes the features and advances in the use of invertebrate animal models in order to study dimorphic fungal infections.

## 2. *Galleria mellonella*

The insect *G. mellonella* (order *Lepidoptera*) is known as the greater wax moth or honeycomb moth as it feeds on the honey, pollen, and wax in the environment and damages honeybee populations and hive products [17]. In 1997, Gotz et al. demonstrated for the first time that larvae of *G. mellonella* were useful to study the entomopathogenic fungi *Metarhizium anisopliae* [18]. Subsequently, Cotter et al. used this model to study *Candida albicans* and *Saccharomyces cerevisiae* infections [19]. Since these studies, the number of publications using *G. mellonella* as a model to study fungi has increased.

The low cost and the ease of maintaining *G. mellonella* in the laboratory and performing experiments are the attractive features of this model. The population presents distinct sexual dimorphism and four different life stages termed as egg, larva, pupa, and adult (moth). The complete life cycle ranges from weeks to months depending on various factors such as temperature, humidity, and food quality [17,20]. In the laboratory, we usually maintain the insects from 27 °C to 30 °C on wax, pollen, and a honey-based food.

In experiments investigating pathogen-host interaction, fungal inoculum can be administered orally, but the most common method involves injection using a syringe (10 µL) through the pro leg. In this case, the exact inoculum concentration is known and the fungus is able to directly enter the hemolymph and further distribute to the larval tissues. Six different types of immune cells termed as hemocytes (prohemocytes, plasmatocytes, granular cells, coagulocytes, spherulocytes, and oenocytoids) are found in the hemolymph of the larvae, and can act against the pathogens similar to mammalian immune cells [21]. Plasmatocytes are the most common hemocytes and together with granular cells, are involved in phagocytosis, nodule formation, and encapsulation [22]. In addition, as an antimicrobial response in *G. mellonella*, infected larvae synthesize melanin that is deposited on the pathogen to encapsulate it leading to hemolymph coagulation and opsonization resulting in black spots or complete black pigmentation [23].

*G. mellonella* has been the most widely used invertebrate model in the study of dimorphic fungi. The reason for this may be the presence of the previously mentioned phagocytic cells and the ability of the larvae to support a wide temperature range including 37 °C, which represents the conditions of human physiology and contributes to maintaining the yeast form of dimorphic fungi and their virulence factors. Because of this, some authors have chosen *G. mellonella* as a model to evaluate the pathogenesis of *Paracoccidioides* spp. [24,25,26,27,28,29,30], *Histoplasma capsulatum* [24], *Sporothrix* spp. [31], and *Talaromyces marneffei (Penicillium marneffei)* [32,33]. However, the *G. mellonella* genome has not been fully sequenced and there are few stock centers that provide larvae bred specifically for scientific purposes, but none that provide mutant strains of larvae. Consequently, the genotypes and breeding maintenance of *G. mellonella* from each laboratory can influence the results of pathogen-host interaction [23]. Despite this, the transcriptome and microRNAs data from *G. mellonella* are available and are important source of information to best understand the host-pathogen interaction [34,35].

After fungal infection in *G. mellonella*, several end-points such as larval survival/death, fungal burden, melanization intensity, fluctuations in hemocyte number and composition, as well as genomic/proteomic changes can be evaluated [36,37]. In addition, antifungal compounds can be also administered using a syringe (10 µL) through the pro leg. Although pharmacokinetic parameters such as distribution volume, clearance, plasmatic proteins bound, and hepatic metabolism cannot be evaluated in this invertebrate model, the efficacy and toxicity of antifungal agents as well as immunomodulatory effects can be examined successfully. Considering these aspects, drugs such as amphotericin B and itraconazole were evaluated against *Paracoccidioides* spp. in *G. mellonella* and demonstrated a dose-dependent benefit to the larvae [27]. It is important to highlight that the induction of an immune response independent of the effect of the antifungal drug can also be observed in *G. mellonella.* This effect was described with caspofungin, from the echinocandins antifungal class. In this study, the efficacy of the treatment was observed with the induction of the immune response and was not related to the response to this antifungal [38]. Based on this, the invertebrate host immune response should be considered during efficacy studies of new antifungal compounds.

## 3. *Caenorhabditis elegans*

The free-living nematode *C. elegans* is commonly found in soil, where there are rotting fruits and stems, and appears to feed on various microorganisms [39]. Since its first use in the laboratory as an *in vivo* model to study development and neurobiology by Sydney Brenner in the 1960s, *C. elegans* has been used to study many aspects of biology including infectious diseases [40].

Some features that make *C. elegans* a useful model include: inexpensive culture, small size, rapid life cycle, transparency, and ease of genetic manipulation [41]. The population is mainly composed of self-fertilizing hermaphrodites, which permits the establishment of homogeneous progenies. The adults are about 1 mm in length and each one can produce a large number of embryos in a 3-day life cycle. The transparency of its body permits visualization of tissues as well as the process of pathogen-host interactions. Furthermore, the nematode has a fully sequenced genome and transgenic strains have been generated by RNA interference (RNAi), which can be delivered systemically by feeding the worms with bacteria and targeting the gene of interest, or by microinjection of DNA (e.g., plasmids and/or PCR products) [42,43,44].

*C. elegans* worms are normally maintained at 15 to 25 °C on plates with nematode growth medium (NGM) with *Escherichia coli* mutant strain OP50 as the food source [45]. Experiments aiming to study pathogen-host interaction involve substitution of *E. coli* with a microbial pathogen (bacterium or fungus), and normally, few hours are sufficient for *C. elegans* to feed on the new microorganism. This process is experimentally easy, but does not allow for determining the exact inoculum concentration [46]. The reproduction of the worms during long experiment can be prevent by the use of Floxuridine, which acts in the prevention of eggs hatching, however the use of this substance could interfere in some experiments during the analysis of gene expression [47]. Another way to prevent *C. elegans* reproduction is the use of transgenic strains as the AU37 (*glp-4*(*bn2*) I; *sek*-*1*(*km*4) X) which is used widely in pathogenesis assays and to evaluate new antifungal candidates. The *glp*-4 mutation generates worms sterile at 25 °C and the *sek-1* mutation worms more susceptible to infection [48,49,50].

In the infection process, the hypodermis and intestine of *C. elegans* are the main tissues exposed to microorganisms and they present similarities with mammalian tissues. The hypodermis comprises a single layer epithelium and a collagen-rich cuticle, whereas the intestine has epithelial cells and actin-rich microvilli to absorb nutrients [51,52].

Furthermore, pathogen-host interaction depends on the immune response. In this aspect, *C. elegans* does not present specialized immune cells such as phagocytes and has no adaptive immunity. On the other hand, it presents several conserved immunological mechanisms of innate immune response that corresponds to the first defense against pathogens in mammalian hosts. In this manner, activation of antimicrobial pathways, such as mitogen-activated protein kinases (MAPKs) and transforming growth factor β (TGF-β), production of reactive oxygen species (ROS), and secretion of antimicrobial molecules occurs in a similar manner between worms and mammals against fungi and bacteria [53,54,55,56]. Thus, *C. elegans* is an advantageous model for studying human infections at a molecular level.

Several fungi including *Candida* species [50,57,58], *Cryptococcus neoformans* [49], and *H. capsulatum* [59] can cause infection in *C. elegans*. However, some fungi such as dimorphic *Paracoccidioides brasiliensis* are not ingested by worms because of features that are discussed below and consequently do not cause fungal infection in this model [60].

Although *C. elegans* has been useful to study the pathogenesis of dimorphic fungi, it is important to consider the issue of temperature. Pathogenesis studies on dimorphic fungi need to be conducted at 37 °C because this is the form responsible for causing disease in the host [61]. The morphological switch from hyphae/conidia to yeast form at 37 °C is indispensable for the upregulation of virulence factors involved in several processes such as nutrients acquisition, adhesion to host tissues, growth and lysis of macrophages, and impairment of cell-mediated immunity among others [62]. However, *C. elegans* worms can be not used at the same temperature as the mammalian host because they are not resistant to high temperatures such as 37 °C for prolonged periods of time—the experiments are usually performed at 25 °C [16,63]. Therefore, the study of dimorphic fungal factors involved in virulence and consequently in pathogenesis needs to be considered in this model, as the temperature changes can promote genome-wide reprogramming of the pathogen [64].

After fungal infection, worm survival can be assessed through of its shape (sinusoidal for live and straight for dead worms) and motility; pathogen burden can be easily and noninvasively analyzed [63]. In addition, antifungal compounds can be added to the wells of plates with infected worms to screen for their toxicity and efficacy. Thus, amphotericin B, terbinafine, and azoles were successfully tested against *Talaromyces marneffei* in this *in vivo* model [65]. The advantage of this model is that a large number of worms can be used to screen large chemical libraries using high-throughput techniques and the results of antifungal efficacy and toxicity can be evaluated simultaneously. *C. elegans* or *G. mellonella* cannot replace the mammalian models in drug discovery, but during screening the best compounds are selected, thus contributing to reduce the number of mammals that will be used subsequently. On the other hand, the disadvantages of this model include difficulty in identifying immunomodulatory compounds and studying pharmacokinetics parameters [46,57].

## 4. *Acanthamoeba castellanii*

*Acanthamoeba* spp. are free-living protozoa, distributed ubiquitously in the soil and aquatic environments. They have a bi-phasic life cycle that includes an active trophozoite stage, in which they exhibit vegetative growth, and a dormant cyst stage with low metabolic activity under harsh environmental conditions [66]. The trophozoites can feed on organic particles as well several microorganisms which often co-exist in the environment [61]. They are often associated with amoebic keratitis in people who wear contact lenses [67].

Spine-like structures termed as acanthopodia are found on the surface of *Acanthamoeba* spp. They contain contractile vacuoles that expel water for osmotic regulation, digestive vacuoles, lysosomes, and glycogen-containing vacuoles [68]. Moreover, although the *Acanthamoeba* genome has not been fully sequenced, due to its polyploid and complex nature, several putative pattern recognition receptors (PRRs) with orthologous functions in the mammalian innate immune systems have been described [69].

Similar to *G. mellonella* and *C. elegans*, *Acanthamoeba* spp., especially *A. castellanii*, has emerged as a simple, rapid, and low-cost model for parasite-host interaction studies. In this aspect, some microorganisms can survive, grow, and evade the amoeba after internalization and this interaction possibly contributes to their transmission to susceptible hosts and/or to their pathogenicity [68,70]. Moreover, the amoeba harboring the pathogen can act as “Trojan horses” protecting it against antimicrobial effectors and environmental conditions and providing conditions for its survival and growth [71].

Several studies have demonstrated the ability of amoebae in selecting virulence characteristics and contributing to the adaptation of pathogens to macrophages [68,70]. Amoebae and macrophages share similarities such as the ability to ingest particles into phagosomes and presence of lysosomal enzymes, which could theoretically make both cells inhospitable to infection by the pathogen; however, the mechanisms developed to escape and survive in the amoeba are similar to those used in macrophages [71]. In addition, experiments with *A. castellanii* can be performed at 37 °C. Thus, these features make this model attractive to explore phagocytosis of different pathogens including dimorphic fungi such as *Blastomyces dermatitidis, Sporothrix schenckii*, and *H. capsulatum* [72].

## 5. Use of *G. mellonella*, *C. elegans* and *A. castellanii* to Study Dimorphic Fungi

**Figure 1:**
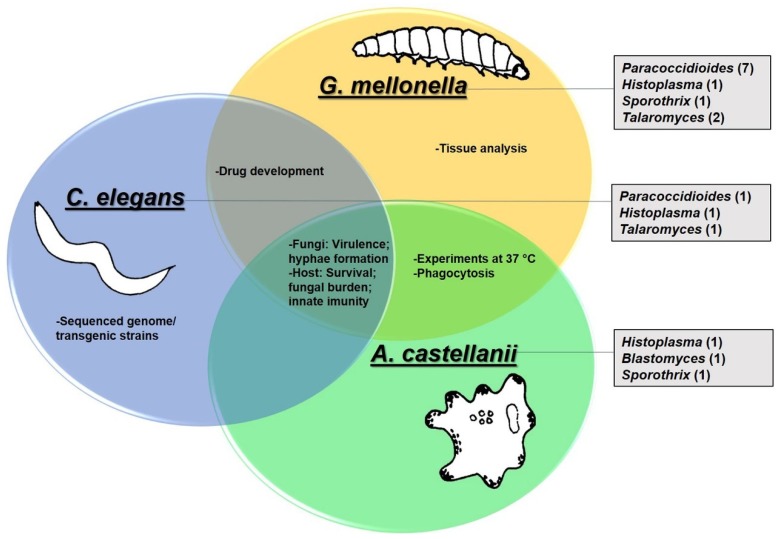
Invertebrate animal models used in the study of dimorphic fungi. Circles show the features and experimental uses specific to each model or common to each other. Rectangles show the dimorphic fungi studied in each model as well as the number of publications in parentheses.

Considering the features of *G. mellonella, C. elegans*, and *A. castellanii*, we report the studies using these models in the context of dimorphic fungi *Paracoccidioides* spp., *Histoplasma capsulatum*, *Blastomyces dermatitidis*, *Sporothrix* spp. and *Talaromyces marneffei (Penicillium marneffei)* in the next sections. The data are summarized in Figure 1.

### 5.1. Paracoccidioides *spp.*

Fungi from the *Paracoccidioides* genus are etiological agents of paracoccidioidomycosis (PCM), an important systemic mycosis endemic on the American continent with high prevalence in South America, especially in Brazil [73,74,75]. In past years, the phylogeny of *Paracoccidioides* spp. was extensively studied and recently, the genus was divided into five species capable of causing disease: *P. brasiliensis*, *P. lutzii*, *P. americana*, *P. restripiensis*, and *P. venezuelensis*, that present consistent genetic, morphological, and geographical differences [76,77,78,79].

**Figure 2:**
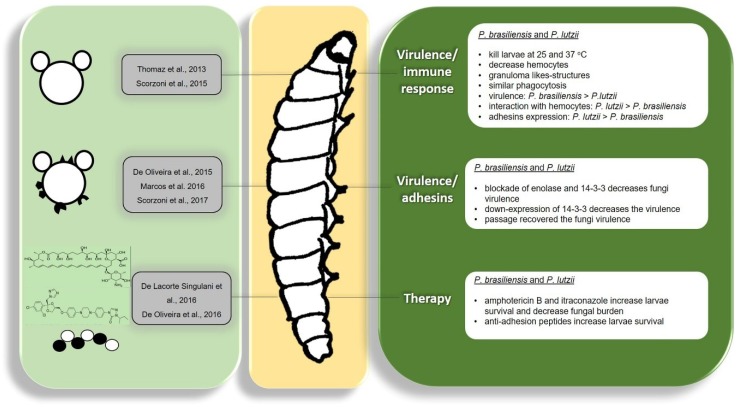
Studies of *Paracoccidioides* spp. with respect to virulence, immune response, and treatment in the *Galleria mellonella* model.

*Paracoccidioides* spp. have developed important virulence factors that facilitate interaction with the host contributing to disease development. During the interaction, *Paracoccidioides* spp. have to adhere and/or invade the host cells and develop strategies to evade the host immune system [80,81,82,83,84,85]. In recent years, knowledge of the virulence factors used by *Paracoccidioides* spp. to interact with the host has increased, and its importance during the infection process has been evaluated using distinct approaches [29,86,87,88,89,90,91]; however, better characterization is required, especially through *in vivo* studies. In this aspect, invertebrate animal models have been recently used to study this important fungus, especially the *G. mellonella* model (Figure 2).

The first report on the use of *G. mellonella* in the study of *Paracoccidioides* virulence was in 2013 by Thomaz et al. The authors validated the use of *G. mellonella* in studying the virulence of *P. lutzii*, as this pathogen was able to cause significant disease in the larvae, killing them at both 25 °C and 37 °C. At 25 °C, the larvae were killed uniformly independent of the inoculum size, whereas at 37 °C, higher inoculum size led to faster larvae killing. Histopathological analysis revealed that *P. lutzii* was able to induce tissue damage in *G. mellonella* and granuloma-like structures were found in the larvae tissue, which is common in mammalian infections of *Paracoccidioides*. The authors also tried to evaluate the colony forming units (CFUs) of infected larvae, but were not able to recover *P. lutzii* from agar plates inoculated with macerated larvae [24].

Scorzoni et al. performed a comparative study between the virulence of *P. brasiliensis* and *P. lutzii* in *G. mellonella*. The comparison showed that both species were able to kill the larvae in a very similar manner suggesting that both species have similar virulence levels in this model; however, the time required to kill 50% of the larvae was shorter for *P. brasiliensis* (3 to 4 days) than for *P. lutzii* (4 to 5 days). Both species were also able to cause severe tissue damage after 4 days of infection, being able to multiply inside the larvae and form granuloma-like structures. The authors also included new parameters in the study of *Paracoccidioides* spp. virulence in *G. mellonella* by investigating the larval hemocyte density after infection and found that infection by both species leads to a decrease in the hemocyte number in a very similar manner [25].

As previously described, during the infection process, *Paracoccidioides* may adhere to or invade host cells, and during this process, may evade the host immune system. Studies on *Paracoccidioides* host cells are necessary because there are many gaps in the existing knowledge, and the *G. mellonella* model can be used for this goal.

Scorzoni et al. evaluated the interaction of *Paracoccidioides* spp. with hemocytes from *G. mellonella* and found that *P. lutzii* interacts in a more effective manner with these cells. After 3 h, a great difference was observed since 48% of *P. lutzii* cells interacted with the hemocytes, whereas only around 14% of *P. brasiliensis* cells did so. However, when phagocytosis was evaluated, the percentage of phagocytosed cells from both species was similar at around 5%, indicating that the larval immune system recognizes both species in a similar manner. The authors also evaluated the *Paracoccidioides* spp. adhesin expression when interacting with *G. mellonella* and found that all the analyzed adhesins were more expressed in *P. lutzii* than in *P. brasiliensis*, which can explain why *P. lutzii* interacted more effectively with hemocytes [25].

Adhesins are molecules produced by *Paracoccidioides* spp. that mediate interaction between the fungi and host cells; some of these molecules are also known to play a role in fungal evasion from the immune system [86,88,89,92,93,94,95].

The importance of these molecules in the interaction of *Paracoccidioides* spp. with the host was evaluated by de Oliveira et al. using *G. mellonella* as the model. The authors evaluated the importance of two major expressed adhesins of *Paracoccidioides*, enolase and 14-3-3, during interaction with mammalian cells *in vitro* and *in vivo*. For this, *P. brasiliensis* and *P. lutzii* cells were treated with antibodies against these adhesins for one hour to block these adhesins and these cells were then used to infect *G. mellonella* larvae. A significant increase in larval survival was observed when infected with *Paracoccidioides* cells previously treated with antibodies revealing the importance of these adhesins to the infection process of *Paracoccidioides* spp. [29].

Marcos et al. also used the *G. mellonella* model to evaluate the importance of the 14-3-3 adhesin by infecting larvae with a 14-3-3 silenced strain of *P. brasiliensis* and observed that the silenced strain is less virulent than the wild type strain. After 24 and 48 h there was a significant decrease in the CFU number of the larvae infected with the silenced strain, revealing the important role of the adhesin 14-3-3 in the virulence of *P. brasiliensis* [30].

Regarding virulence studies, *Paracoccidioides* spp. and other dimorphic fungi are strongly affected by *in vitro* storage and successive subcultures; one way to restore their virulence characteristics is to recover the fungi from infection in mammalian hosts, which generates ethical issues since the use of these animals should be minimized [96]. In this manner, Scorzoni et al., evaluated whether *G. mellonella* could be used as an animal model capable of restoring the attenuated virulence of *P. brasiliensis*. Upon comparing subcultured *P. brasiliensis* recovered from mice and *G. mellonella*, they observed that the strains in both cases presented an increased ability to interact with the host. Moreover, there was an increase in the expression of all tested adhesins (enolase, Gp43, triosephosphate isomerase and 14-3-3) of the fungus after passage in mice or in *G. mellonella*. These results indicate that the *G. mellonella* model can be successfully used to reactivate the virulence factors of *Paracoccidioides* with the advantage of rapid isolation of fungi compared to that in mice (4 days for *G. mellonella*; 30 days for mice), while avoiding the use of a mammalian model for this goal [26].

PCM presents two clinical manifestations: 1) the acute/subacute (“juvenile” form), the most severe clinical manifestation, that mainly affects children and young adults of both genders comprising 5 to 25% of cases involving the lymph nodes, liver, spleen, and bone marrow; and 2) the chronic (“adult” form) responsible for 74 to 96% of the cases, that mainly affects adult men over 30 years of age, involving lesions in the lungs in 90% of the cases, but also affecting the mucosa of the upper aerodigestive tract and the skin [73,97,98]. Itraconazole is the first-choice drug to treat PCM, but the availability of this drug in endemic areas is limited; therefore, combination of sulfamethoxazole/trimethoprim is a useful option; on the other hand, amphotericin B is the drug choice in severe cases [75,84,97]. However, as in other mycosis, the treatment period is long, the drugs present toxicity to patients, and several cases of relapses are reported. This indicates the necessity of investing in the research and development of new therapeutic strategies.

The first study that used an invertebrate animal model to evaluate antifungal treatment efficacy for paracoccidioidomycosis was conducted by De Lacorte Singulani et al.; they evaluated the efficacy of amphotericin B and itraconazole in treating of *G. mellonella* larvae infected with *P. brasiliensis* and *P. lutzii*. The authors observed that treatment with both drugs increased larvae survival when infected with both *Paracoccidioides* species in a similar manner and observed a dose-response effect. Moreover, CFUs analysis showed a fungicidal effect of amphotericin B whereas itraconazole demonstrated a fungistatic effect against *Paracoccidioides* spp. By histopathological analysis, the authors could observe the presence of both species mainly under the cuticle and in the peripheral adipose body of the larvae. They also observed fungal aggregates with hemocytes recruitment and melanization spots, and treatment with both antifungals resulted in significant reduction of the aggregate size. In this study, the authors concluded that *G. mellonella* is a valid model to study the response of *Paracoccidioides* spp. to different antifungal agents and could be an important tool in screening new antifungal molecules and compounds against these fungi [27].

The search for new alternatives in therapies generally includes screening of a great number of molecules or active compounds by *in vitro* approaches followed by *in vivo* studies, which could require the use of a great number of animals. Thus, use of invertebrate animal models can become an alternative to avoid using a large number of animals in mammalian models.

In this manner, De Oliveira et al. used a phage display library to screen peptides that could prevent the adhesion process of *Paracoccidioides* spp. to the host since this process is essential for infection by these fungi. After *in vitro* screening, the authors selected four peptides that do not have antifungal activity, but inhibit the adhesion of *Paracoccidioides* spp. to pneumocytes and distinct extracellular matrix components; the behavior of these peptides during an infection of *Paracoccidioides* was then investigated. As the use of mammalian models to test these four peptides would require a large number of animals, they decided to screen this activity using the *G. mellonella* model. For this, larvae were treated with each peptide prior to infection with *P. brasiliensis* and *P. lutzii* and larval survival was assessed. All the peptides showed increased survival in the treated larvae, but only one peptide, called peptide 4, was able to efficiently increase the survival for both species. In addition, the same peptide was able to increase the production of hemocytes that can help the larvae to fight the infection; thus, this peptide was selected to be tested in mammalian models in future studies [28].

All these works highlight the utility of *G. mellonella* as a model to study *Paracoccidioides* spp. and suggest an increase in the number of studies using this animal model to study PCM in the future, opening possibilities to increase our knowledge of this important mycosis.

Among other invertebrate animal models, Scorzoni et al. recently evaluated the possibility of using the nematode *C. elegans* to study fungi of the *Paracoccidioides* genus. In this study, the authors observed that neither *P. brasiliensis* nor *P. lutzii* could infect *C. elegans* due to the irregular shape and size of the fungi, which hampers their ingestion by the nematode. However, the authors observed that simple exposure to the fungi triggers activation of immune responses in the nematode by increasing the expression of antimicrobial peptide genes, which in some cases, is exacerbated when the nematodes are in contact with *P. brasiliensis* than with *P. lutzii*, suggesting that each species could demonstrate a distinct pattern of nematode immune response activation [60]. This is the first report on the use of *C. elegans* to study *Paracoccidioides* and the exposure of the nematode to the fungi causing immune response activation, even without ingestion; this should thus be further explored in future studies.

### 5.2. Histoplasma capsulatum

The systemic mycosis, histoplasmosis, is endemic in certain areas of America, Africa, and Asia and *H. capsulatum* is one of the causative organisms [99]. *H. capsulatum* can infect macrophages, resist antimicrobial defenses, and proliferate as an intracellular pathogen; to deal with the nutritional limitations found in the phagosome environment, it uses different skills such as *de novo* biosynthesis pathways related to production of compounds such as uracil, riboflavin, and pantothenate [100].

Thomaz et al. [24] for the first time demonstrated the possibility of using the *G. mellonella* model of infection for *H. capsulatum*. In mammalian models, the size of the inoculum influences the disease evolution, but in *G. mellonella*, there is no relationship between the number of yeasts and time required to kill the larvae at 25 °C and 37 °C for *H. capsulatum*, suggesting that the pathogen evokes protective responses once the lower inoculum concentration leads to faster killing of the larvae [24].

Production of α-glucan is essential to the virulence of *Histoplasma* yeast as its presence on the fungal cell wall masks the β-glucan layer, blocking interaction with the mammalian receptor dectin-1 [101]. Comparing the virulence profile of two *H. capsulatum* strains, G184AR and G217B, Thomaz et al. [24] demonstrated that at both evaluated temperatures, G184AR was more virulent than the G217B strain, which does not present α-glucans on its cell wall. Therefore, the results indicate that *G. mellonella* has the ability to recognize β-glucan, and is a useful model for the initial elucidation of possible mechanisms related to the immune evasion ability of pathogens.

In addition, *H. capsulatum* could induce larval melanization in a dose-dependent manner within hours after infection (6 h) at 25 °C and 37 °C, but this response was more evident at 37 °C, and this response alone was not enough to protect the larvae as intensely melanized larva succumbed to death [24].

The yeast phase of the fungus was used for *G. mellonella* infection and hyphae transition during the course of infection at 25 °C was not visualized [24] as this process takes several weeks. Therefore, studies using conidia instead yeasts need to be performed in this model, at this specific temperature, to investigate the transformation of the fungus. More recently, Cordero et al., [102] evaluated the interactions of *H. capsulatum* and *Cryptococcus neoformans* in a synchronous infection by incorporating *in vivo* glycans (formed by glucuronoxylomannan and chitin-like molecules) from *C. neoformans* in *H. capsulatum* and demonstrated increased virulence in the latter, which was directly related to the increase in the concentration of the incorporated glycan, in the *G. mellonella* model. This demonstrated that interaction with both fungi could modify the ability of *H. capsulatum* to interact with the host, being relevant to hosts co-infected with these pathogens.

Similar to *G. mellonella*, *C. elegans* is a useful model to study *H. capsulatum*, as it can ingest the pathogenic yeasts. After ingestion, the integrity of the fungus could be confirmed through the use of yeast forms of *H. capsulatum* expressing a GFP-tag; the infection resulted in high lethality of worms, as first described by Johnson et al., [59]. They described the use of a *C. elegans* model for *H. capsulatum* to evaluate the early stages of pathogenesis. Exposition to the virulent strain (*H. capsulatum Nam1*) caused the death of 65% and 90% of the worms after 24 h and 48 h, respectively. Moreover, the short time required for *H. capsulatum* to kill the worms allows the assay to be performed in a single plate within 8-12 h of fungus infection, eliminating the adult worm transference for separation from the newborn progeny. Additionally, they demonstrated that the nematode model can be adaptable to virulence assays as the exposure of *C. elegans* to the *H. capsulatum ura*^-^ strain (lacking *de novo* uracil biosynthesis) does not cause substantial lethality. The death rate was lower than 10% at 24 h and 23–28% at 48 h, being less toxic compared to the virulent and heat killed strains, confirming the importance of physiological fitness and virulence of the yeasts [59].

Inactivation of the URA5 gene results in pyrimidine biosynthetic pathway disruption and uracil auxotrophy, and uracil-auxotrophic yeasts of *H. capsulatum* were avirulent in macrophages [103] as the phagosome environment does not contain uracil [100]. Similarly, the low lethality of *C. elegans* exposed to *H. capsulatum ura^-^* strain suggests that this model can simulate the virulence observed in mammalian hosts [59].

Although *C. elegans* is a powerful model to study *H. capsulatum* pathogenesis, it is important to consider that the temperature of experiments with this model (15–25 °C) differs from the ideal temperature of the yeast phase of dimorphic fungi as described previously. In addition, the absence of professional phagocytes in *C. elegans* represents a disadvantage of this model for important studies related to phagocytosis of *H. capsulatum* that exhibits a high avidity for intracellular residence [104].

The interaction of *H. capsulatum* and the *Acanthamoeba castellanii* model was also studied and phagocytosis of this pathogen by *A. castellanii* and J774.6 macrophages was compared [72]. During the amoeba-*H. capsulatum* interaction, fungal cells are phagocytosed and internalized into membrane-bound vacuoles, with one or more fungal cells or separated. Once phagocytosed, *H. capsulatum* was able to promote the death of amoebas after 24 h. To explore the interaction of *A. castellanii* with the fungal form found in the environment, and therefore, the one that the amoeba would probably come into contact with at some point in life, *H. capsulatum* conidia were incubated with the amoeba. After 24 h of incubation, the conidia were internalized and some of these were converted to yeast cells, demonstrating that this model can simulate the conversion that occurs in patients as morphological transformations are induced by temperature and environmental changes.

In addition, there was a significant difference in *H. capsulatum* internalization, with 81% to amoeba against 70% to macrophage phagocytosis [72]. After 24 h of incubating *H. capsulatum* with *A. castellanii* at 37 °C, the yeast cells presented a great number of hyphae and pseudohyphal cells, as reported in response to macrophages [105,106]; moreover, the amoeba can be used as a nutritional source, as the interaction contributes to fungal growth [72].

Finally, *H. capsulatum* contact with *A. castellanii* alters the virulence phenotype of the fungi, as the avirulent stain of *H. capsulatum* after passage in *A. castellanii* acquires the ability to persist *in vivo* [72].

### 5.3. Blastomyces dermatitidis

*B. dermatitidis* is a human pathogen of the lungs that causes disseminate disease (blastomycosis) in healthy and immunocompromised individuals, and is mainly found in areas of the United States and Canada [107].

The interaction of *B. dermatitidis* and *Acanthamoeba castellanii* was described by Steenbergen et al. [72]. After 24 h of incubation at 37 °C, *B. dermatitidis* yeast cells presented a great number of hyphae and pseudohyphal cells in the *A. castellanii* model, as reported in response to macrophages [105,106]. In addition, *B. dermatitidis* could promote the death of amoebas after 24 h (ranging from 25 to 37% of death) and showed continuous death at 48 h. *B. dermatitidis* showed a very low phagocytosis rate when compared to *H. capsulatum*, but a similar rate when comparing the rate obtained with amoeba and macrophages (± 2%), possibly due the slower replication rate of this fungus [108]. Although the amoeba-*B. dermatitidis* interaction resulted in low phagocytosis of yeast cells where the vast majority was adhered to the amoeba membrane, the process resulted in mitochondrial swelling and reduced cytoplasmic electron density in the amoeba [72]. This characteristic was not observed in the model with *H. capsulatum*, suggesting that *B. dermatitidis* leads to cytotoxicity in amoebae similar to the extracellular toxicity in macrophage through adhesion and expression of virulence factor *Bad1* (*Blastomyces* adhesin-1) at the cell surface [109].

Taken together, the data with *H. capsulatum* and *B. dermatitidis* show that *A. castellanii* is a useful model for studying dimorphic fungi as the exposure to amoebae yields data of fungal virulence similar to those in mammalian models [72].

### 5.4. Sporothrix *spp.*

Sporotrichosis is a fungal infection with worldwide distribution, more prevalent in tropical and subtropical regions, with increasing incidence in the last two decades, caused by the *Sporothrix* complex. *S. schenckii sensu stricto*, *S. brasiliensis*, and *S. globosa*, are the most relevant clinical species; however, *S. luriei*, *S. mexicana*, *S. pallida* (or *albicans*), and *S. chilensis* are environmental species that are described as opportunistic pathogens in immunosuppressed patients [110,111,112].

Infection usually occurs by traumatic injury from contaminated material or transmission through bites or scratches from infected animals and the disease may present different clinical manifestations, from superficial to systemic mycosis, depending on the host immune system and virulence of the fungus, which can vary among species. [31,113,114]. Therefore, *in vivo* models are important to study the features of different species with respect to their virulence and host-pathogen interactions.

*Galleria mellonella* had been reported as being a suitable model to study *Sporothrix* complex infection. Clavijo-Giraldo et al. [31] analyzed larval survival upon infection with conidia, germlings, or yeast-like forms of *S. schenckii sensu stricto* 1099-18 ATCC MYA 4821 and *S. brasiliensis* 5110 ATCC MYA 4823, and demonstrated that *Sporothrix* spp. infect and kill larvae in a dose-dependent manner.

In addition, infection with conidia and germling cells from both strains did not show a significant difference in larval survival at all the concentrations tested. However, in the infection with yeast-like cells, which is the form associated with the disease, a significant increase in the mortality rate due to infection with *S. brasiliensis* was observed when compared with *S. schenckii sensu stricto*, suggesting that *S. brasiliensis* is more virulent in *G. mellonella*, as previously observed in a murine model [31,115].

The virulence of yeast-like cells from different strains of *S. schenckii* sensu *stricto* and *S. brasiliensis* was also compared in *G. mellonella.* The *S. schenckii* sensu *stricto* 1099-18 ATCC MYA 4821, Ss39 and Ss47 strains, previously characterized as low virulent strains in the mouse model [115,116], and the strain SS-B02, without the virulence degree reported but virulent in the mouse model for systemic infection [117], were used to infect larvae. Significant differences in mortality rates were observed, where the SS-B02 and Ss39 were more virulent in *G. mellonella* than the reference strain and Ss47, which according to the authors seem to have similar virulence degrees [31]. The results of this study correspond with the findings in mouse models, except for the Ss47 and Ss39 strains, which were reported to have the same virulence degree in a mouse model [116]. This was justified by the use of different concentrations of carbon source in the medium, which could affect the virulence of the strain [31].

In the study of *S. brasiliensis* virulence in *G. mellonella*, the strains 5110 ATCC MYA 4823, Ss54, HUPE 114158, HUPE 114500 and UFTM01 were used and the strain 5110 ATCC MYA 4823 killed all the larvae faster than the other strains, demonstrating a higher virulence in this model, followed by 5110 ATCC MYA 4823, that showed a similar mortality rate, and HUPE 114500 and UFTM01 strains, which were demonstrated to be less virulent compared to other strains [31]. These results were in line with a previous study on subcutaneous infection in mice, where 5110 ATCC MYA 4823, Ss54, and HUPE 114158 were characterized as high virulent strains, and HUPE 114500 and UFTM01 presented intermediate virulence [115]. These results indicate that *G. mellonella* is a suitable model to study *Sporothrix* spp. virulence.

Another non-conventional model proposed to study *Sporothrix* is *A. castellanii*, as its phagocytic features can be compared to macrophage actions during infection and has been demonstrated as a host of bacterial and fungal pathogens in the environment, which suggests that this interaction could influence the virulence factors in pathogens found in soil [118,119,120,121], such as *Sporothrix* spp.

Steenbergen et al. [72] compared the interactions of *S. schenckii* with *A. castellanii* and macrophages (J774.16) and found that the amoeba phagocytoses the yeast cells at a rate similar to macrophages. Electron microscopy analysis showed that several fungal cells may be phagocytosed and enclosed in membrane-bound vacuoles. After 24 h of infection, an increased number of hyphal or pseudohyphal cells was reported.

In the presence of *A. castellanii, S. schenckii* showed a significant increase in CFU, of almost 35-fold when compared to the fungus in PBS, after 48 h. In addition, *S. schenckii* killed the amoeba cells, causing significant amoeba death within 24 h and after 48 h, 25-37% of amoeba cells were dead [72].

### 5.5. Talaromyces marneffei (Penicillium marneffei)

*Talaromyces marneffei*, previously known as *Penicillium marneffei*, is one of the most important thermally dimorphic fungi that causes systemic mycosis called talaromycosis, in Southeast Asia [122]. The infection is usually associated with AIDS patients, as an opportunistic infection, and is considered the third most prevalent opportunistic infection associated with AIDS in some regions, such as Thailand, Hong Kong, and southern China [123,124,125,126]. However, an increase in the incidence of talaromycosis in HIV-negative patients has also been reported, mostly associated with patients under immunosuppressive therapies or due to other immunodeficiency syndromes [127,128,129].

Huang et al. [65] studied the use of *C. elegans* as a model of infection in solid and liquid medium and reported that *T. marneffei* can infect and kill the nematodes with similar outcomes. However, it was also observed that nematode death occurs earlier in solid medium than in the liquid, probably due to greater exposure of *C. elegans* to the fungus.

In this study, two strains of *T. marneffei*, SUMS0486 and SUMS0570, were evaluated; it was found that the SUMS0570 strain, which produces a red pigment as a secondary metabolite, could kill the nematodes faster. In addition, the authors observed that the intestine of *C. elegans* was extended in the beginning of the infection (4 h) and in the following 72 h of the infection it was gradually filled with the red pigment. These features were not observed during SUMS0486 infection; therefore, it was suggested that the red pigment might be involved in *T. marneffei* virulence in this model [65].

During the infection in *C. elegans*, hyphal formation in the nematode intestine was reported the and the strain SUMS0570 hyphae destroyed and penetrated about 50% of the infected *C. elegans*. Hyphae were also observed in the nematodes infected with SUMS0486, but this was only reported in 25% of the infected *C. elegans* [65].

In addition, Huang et al. [65] tested five antifungals (amphotericin B, terbinafine, fluconazole, and itraconazole and voriconazole) to evaluate the use of this model to study antifungal agents against *T. marneffei* infection and reported that all antifungals could increase the survival of nematodes; the best outcomes were with voriconazole and amphotericin B treatment (60% viability), and fluconazole presented the lowest survival rate (35%). Therefore, the authors concluded that *C. elegans* is a suitable model for studying *T. marneffei* infection, and suggested that hyphae formation and secretion of the red pigment might be involved in the pathogenesis. Thus, this model could be used for screening antifungal compounds against this fungus.

The use of *G. mellonella* was also evaluated to study *T. marneffei* by Huang et al. [33]. The ability to infect larvae was assessed by injecting different concentrations of *T. marneffei* wild type strain F4 conidia and a dose-dependent effect on the death rate of larvae was observed. As discussed before, the advantage of this model is the possibility of performing experiments at a range of temperatures, therefore simulating the environmental and mammalian host temperature. This study reported that infection leads to larval melanization and death. Experiments were conducted at both 25 °C and 37 °C and no difference in larvae killing was observed between these temperatures, indicating that both mycelia and yeast-like forms can cause infection in larvae. However, on the fourth day after infection, a higher percentage of larvae (70%) were dead at 37 °C compared to those at 25 °C (50%).

The virulence of *T. marneffei* was also evaluated in this study, using the same strains as those from the *C. elegans* study [65], SUMS0486 and SUMS0570. The red pigment producing strain, SUMS0570, was found to kill *G. mellonella* larvae faster than SUMS0486 when the experiment was conducted at 25 °C. However, no differences were observed in *G. mellonella* death between the two infected groups at 37 °C, which would indicate the involvement of the red pigment in *T. marneffei* pathogenesis, demonstrating that *G. mellonella* can be used in studying the infection and virulence of *T. marneffei* [33].

This study also reported that *G. mellonella* could phagocytose *T. marneffei* and therefore, this model can be used to study the immune response against the infection and *T. marneffei*-host interactions [33].

This approach was used by Suwunnakorn et al. [32], for the *in vivo* characterization of *rttA* gene from *T. marneffei.* The *rttA* gene from *T. marneffei* encodes a histone acetyltransferase enzyme (HAT) that is involved in growth and development especially in the mycelial phase and in DNA damage repair, and the expression of this gene is suggested to be important for viability maintenance during stress response. The comparison of *T. marneffei* wild type strain F4, a mutant strain I133 (ΔrttA) and a genetically complemented transformant, CR5, (ΔrttA+rttA) was performed in *G. mellonella* and it was demonstrated that the ΔrttA mutant was less virulent in this model, showing a significant decrease in mortality in the experiment conducted at 37 °C. Analysis of *G. mellonella* tissue showed that phase transition is affected in the mutant, indicating the role of *rrtA* gene in the virulence and phase transition of *T. marneffei*.

## 6. Conclusions

Invertebrate animal models have been well accepted by the scientific community, which is evident from the growing number of publications using these models. They represent a great strategy for *in vivo* assays for fungal infections because of the speed of the tests, the price, and the number of individuals used for each assay. Considering dimorphic fungi, invertebrate models have been good models to evaluate virulence factors, to compare the virulence between strains or species, to assess immune responses, and the efficacy and toxicity of antifungal agents. However, it is important to remember that no available invertebrate model has been able to completely substitute vertebrate models.
